# Does obesity cause chronic inflammation? The association between complete blood parameters with body mass index and fasting glucose

**DOI:** 10.12669/pjms.331.11532

**Published:** 2017

**Authors:** Tuba Tulay Koca

**Affiliations:** Tuba Tulay Koca, Medical Doctor, Physical Medicine and Rehabilitation Clinic, Health Minister, Malatya State Hospital, Malatya, Turkey

**Keywords:** Complete blood count, Body mass index, Fasting glucose, Inflammation, Obesity

## Abstract

**Objective:**

This study aimed to determine the relationship of complete blood count (CBC) parameters and derivates with fasting blood sugar and the body mass index.

**Methods:**

This was a prospective, observational clinical study. Hospitalized patients who received a physiotherapy program in the Physical Medicine and Rehabilitation Clinic between March and June 2016 were included. The age, height, weight, body mass index (BMI), fasting blood glucose, erythrocyte sedimentation (ESR), C-reactive protein, and CBC parameters (leukocytes, platelets, neutrophil, lymphocytes, and monocytes) and red cell distribution width, platelet distribution width, neutrophil–lymphocyte ratio (NLR), and platelet–lymphocyte ratio of the patients were recorded. The relationship between the BMI, fasting glucose, and CBC parameters and derivates were investigated. Patients were divided into groups based on BMI: BMI≤25 kg/m^2^, normal; BMI=26–30 kg/m^2^, overweight; and BMI>30 kg/m^2^, obese. A P value>0.005 was considered statistically significant.

**Results:**

A significant difference in the lymphocyte count, ESR, and NLR values was observed among the three groups (P=0.011; P=0.021; P=0.04). A significant difference in NLR was found between groups 1 and 3 (P=0.04). Between groups 1 and 3, a significant difference in platelet count was noted (P=0.013). On dividing the patients into two groups: normal and overweight/obese, a significant difference in lymphocyte count, glucose, and ESR values was observed (P=0.038; P=0.05; P=0.013). The lymphocyte count, ESR, and glucose values were found to be higher in the overweight group. According to Spearman’s correlation analysis, the BMI and NLR values were found to be negatively correlated (P=0.029; r=.145); however, the lymphocyte count and ESR values were positively correlated (P=0.009; r=.173); (P=0.013; r=.182).

**Conclusion:**

This study found a negative correlation between the NLR and BMI values and a lower NLR value in the obese group compared with the normal group. The overweight group showed a higher lymphocyte count, thereby confirming the positive correlation of lymphocyte count with BMI. A comprehensive clarification of the mechanisms underlying the relationship between obesity and inflammation may allow developing treatment strategies to reduce the negative effects of obesity.

## INTRODUCTION

Obesity results in cardiovascular disease and an increase in the risk of type 2 diabetes. The global increase in obesity prevalence led to an increase in the frequency of metabolic syndrome, which resulted in the need to understand the underlying mechanisms.^1^

Many studies associated the complete blood count (CBC) and the resulting ratios with the neutrophil–lymphocyte ratio (NLR), platelet–lymphocyte ratio (PLR), and some diseases and their clinical courses. A study determined the fasting blood glucose, body mass index (BMI), and effect on blood parameters of obesepatients.^2^ The present study aimed to evaluate the potential impact of BMI and fasting blood glucose on the CBC parameters and derivatives.

## METHODS

This was a prospective, observational clinical study. Hospitalized patients who participated in a physiotherapy program in the Physical Medicine and Rehabilitation Clinic between March and June 2016 were included in this study. The age, height, weight, BMI, fasting blood glucose, erythrocyte sedimentation (ESR) (0–20 mm/h), C-reactive protein (CRP) (0–0.8mg/dl) and CBC parameters (leukocytes, platelets, neutrophil, lymphocytes, and monocytes), red cell distribution width (RDW), platelet distribution width (PDW), and derivates (NLR, PLR) of the patients were recorded. The relationship between the BMI, fasting glucose, and CBC parameters and derivates was investigated. Patients were divided into groups based on BMI: BMI ≤25 kg/m^2^, normal; BMI=26–30 kg/m^2^, overweight; and BMI >30 kg/m^2^, obese. The patients were also divided into two groups based on BMI: normal and overweight/obese. The fasting blood glucose, BMI, and CBC parameters were compared.

The statistical analysis was performed using IBM SPSS for Windows, version 21.0 software (IBM Corporation, NY - USA). Descriptive data were presented as mean ± standard deviation and median scores. The Mann–Whitney U test and Kruskal–Wallis test were used to analyze abnormally distributed data. The Spearman’s correlation analysis was used to analyze the level of correlation between variables. The coherence of variables to normal distribution (normality) was analyzed using the Kolmogorov–Smirnov test. A p-value of <0.05 was considered statistically significant.

## RESULTS

This study included 158 women (68.4%) and 73 men (31.6%) (Table-I).The mean age of the patients was 57.1±16.3 years; the mean value of BMI was 27.8±5.1 kg/m^2^. The mean value of ESR of patients included in this study was 25.3±14.8 mm/h; the mean value of CRP was 1±8 g/dL, and the mean value of fasting glucose was 121.6±62.2 g/dL (Table-II). The distribution of these values according to groups is given in (Table-III).

**Table-I:** Age, BMI, ESR, CRP, and FBG values.

	Age	ESR	BMI	CRP	FBG
N	230	230	230	230	230
Mean	57.32	25.3194	27.8171	1.0367	121.67
Standard deviation	16.248	14.86421	5.20387	8.04780	62.258

BMI: body mass index; CRP: C-reactive protein ESR: erythrocyte sedimentation rate; FBG: fastıng blood glucose.

**Table-II:** ESR, fasting glucose, and CRP values of all groups

	Groups	ESR	Glucose	CRP
Normal	Mean	20.7500	114.71	1.7666
	N	68	76	76
	Standard deviation	13.26017	53.612	12.82142
25-30 overweight	Mean	27.4857	133.08	0.6723
	N	70	65	65
	Standard deviation	14.77046	79.483	1.52974
>30 obese	Mean	28.3208	117.60	0.4481
	N	53	48	54
	Standard deviation	15.76239	45.996	0.58362
Total	Mean	25.3194	121.76	1.0367
	N	191	189	195
	Standard deviation	14.86421	62.410	8.04780

CRP: C-reactive protein; ESR: erythrocyte sedimentation rate.

**Table-III:** CBC parameter and derivative values

	Minimum	Maximum	Mean	Standard Deviation
Leukocyte	3.920	17.450	7.54662	2.190777
Platelet	142.000	591.000	288.37719	75.977204
Neutrophil	1.400	82.100	5.27508	6.367838
Lymphocyte	0.150	11.400	2.12497	0.935861
Monocyte	0.060	6.400	0.52342	0.673543
RDW	10.700	48.000	14.44348	4.267602
PDW	7.800	18.900	15.85618	0.980572
NLR	0.57	17.48	2.7396	2.16918
PLR	0.26	17.40	1.5844	1.23967

NLR: Neutrophil–lymphocyte ratio; PDW: Platelet distribution width; PLR: Platelet–lymphocyte ratio; RDW: Red cell distribution width.

The CBC parameter and derivative values of the patients are given in Table-IV). The distributions of CBC parameters and derivates according to groups are given in (Tables-V). A significant difference in the lymphocyte count, ESR, and NLR values was found among the three groups (P=0.011; P=0.021; P=0.04). The average lymphocyte count and ESR values were higher in the obese and overweight groups, and the NLR value was lower in the obese group.

**Table-IV:** Distribution of CBC parameters.

Group		Leukocyte	Platelet	Neutrophil	Lymphocyte	Monocyte
Normal	Mean	7.54602	273.78409	5.61942	2.05822	.53434
	N	88	88	89	89	89
	Standard deviation	2.415059	63.062357	8.479803	1.229092	.652518
25–30 overweight	Mean	7.59410	293.87179	5.50139	2.05316	.58861
	N	78	78	79	79	79
	Standard deviation	2.073063	88.134543	5.939608	.692881	.907481
>30 obese	Mean	7.48774	302.17742	4.49242	2.31226	.42468
	N	62	62	62	62	62
	Standard deviation	2.027491	73.925422	1.587071	.667792	.142239
Total	Mean	7.54662	288.37719	5.27508	2.12497	.52342
	N	228	228	230	230	230
	Standard deviation	2.190777	75.977204	6.367838	.935861	.673543

**Table-V:** CBC parameter and derivative distribution.

Group		PDW	RDW	NLR	PLR
Normal	Mean	15.71011	14.36180	2.9980	1.5926
	N	89	89	89	88
	Standard deviation	1.468073	4.333682	2.30528	0.82140
25–30 overweight	Mean	15.93824	14.14937	2.9672	1.7298
	N	79	79	79	78
	Standard deviation	0.479646	2.189654	2.55807	1.88659
>30 obese	Mean	15.96129	14.93548	2.0788	1.3897
	N	62	62	62	62
	Standard deviation	0.393952	5.897376	1.01105	0.44512
Total	Mean	15.85618	14.44348	2.7396	1.5844
	N	230	230	230	228
	Standard deviation	0.980572	4.267602	2.16918	1.23967

NLR: Neutrophil–lymphocyte ratio; PDW: platelet distribution width; PLR: Platelet–lymphocyte ratio; RDW: red cell distribution width.

A significant difference in the NLR value was found between groups 1 and 3 (P=0.04). Between groups 1 and 3, a significant difference in platelet count was noted (P=0.013). The platelet count was found to be higher and the NLR lower in the obese group. No statistically significant difference was found in the other blood parameters.

***On dividing the patients into two groups***

Normal and overweight/obese, a significant difference in lymphocyte count, glucose, and ESR values was observed (P=0.038; P=0.05; P=0.013). The lymphocyte count, ESR, and glucose values were found to be higher in the overweight group. No statistically significant difference was found in the other blood parameters.

According to Spearman’s correlation analysis, the BMI and NLR values were negatively correlated (P=0.029; r=.145); however, the lymphocyte count and ESR values were positively correlated (P=0.009; r=.173); (P=0.013; r=.182). The BMI and platelet count were not correlated.

## DISCUSSION

The incidence of overweight and obesity is gradually increasing in developed and developing countries around the world. Obesity is an important cause of cardiovascular (CVS) diseases, musculoskeletal system diseases, and malignancies. Also, it is one of the preventable causes of death. Metabolic syndrome is a common and complex disorder often seen together with dyslipidemia, hypertension, and insulin resistance.

Obesity is related to many diseases including different types of cancers and is a major public health concern. Obesity has a negative effect on the development and prognosis of breast cancer. The pathophysiological mechanisms underlying the relationship between obesity and cancer are still subject to research.^3^ See comment in PubMed Commons below

Evidence shows that obesity is related to insulin resistance and low-grade chronic inflammation. It has also been proven that obesity is part of many complications. Macrophages accumulated in the fat tissue of obese individual’s have significance in the development of obesity-related inflammation. The number of macrophages in the fat tissue is strongly related to the person’s weight, BMI, and total body fat. Measures to reduce the number of macrophages or inflammatory properties reduce systemic inflammation and increase insulin sensitivity.^4^

The underlying mechanisms are important for gerontology, since obesity, insulin resistance, and inflammation are also related to aging. Although the reduction in insulin sensitivity in obese people is related to various molecular and cellular mechanisms, the pathogenesis of obesity-related insulin resistance is still unclear. Recent studies focus on the fact that inflammation is induced by macrophages in the adipose tissue; however, the involvement of other organs (liver, muscles, and pancreas) also needs to be considered.^5^

Pre-diabetes contributes to the development of cardiovascular disease and is associated with central obesity, inflammation, oxidative stress, and endothelial dysfunction. A study by Holvoet P et al.^6^ found that the metabolic syndrome was related to a high fraction of oxidized low-density lipoprotein. It is known that metabolic syndrome is closely related to insulin signaling, oxidative stress, inflammation, and atherosclerosis.^7^

Abdominal obesity is due to an increased oxidative stress and decreased nitric oxide associated with endothelial dysfunction. Perivascular adipose tissue–induced proinflammatory cytokines in obese individuals target the vasculature, which is a source of low-grade inflammation and oxidative stress. This contributes to endothelial dysfunction.^8^

Zhao et al.^9^ assessed the risk of diabetes with anthropometric measurements and found that women with diabetes and pre-diabetes have increased BMI, waist–hip ratio, and waist–height ratio.

Acute-phase protein markers (CRP and ESR) are insufficient to show disease activity, and only few specific biomarkers help in controlling the disease. Since acute-phase proteins increase only as an indirect result of the local inflammatory process, they are insufficient to show a systemic inflammatory response. Also, the ESR and CRP values can be affected by new infections and are nonspecific in estimating inflammation.^10^-^12^

Research on neutrophil and subgroups and their relationship with inflammation has increased in recent years. Many studies have found a positive correlation between RDW, CRP, and ESR.^13^,^14^ High RDW has been associated with undesirable consequences in various clinical situations. The mechanism behind it is not known. Metabolic syndrome increases the chances of CVS disease and causes death. The extensive cohort study by Laufer et al.^15^ found that an RDW value of>14%was independently associated with metabolic syndrome and long-term mortality. A study of Rodrigez-Carrio J et al.^16^ found that RDW was associated with a reduction in endothelial progenitor cells and vascular repair insufficiency and related to increased levels of various mediators. It was concluded that RDW was an important indicator of CVS diseases.

The present study compared CBC parameters and derivatives with the BMI and fasting glucose levels. When divided the patients into normal and overweight/obese, it was found that only the NLR, lymphocyte count, ESR, and glucose values were of statistical significance and correlated with BMI. However, the platelet count was found to be statistically significantly different between groups one and three. In other words, the more a person gains weight, the more the increase in lymphocyte count, platelet count, and ESR values. The NLR value, however, decreases. No significant difference was found in the other parameters.

## CONCLUSION

The present study found a negative correlation between the NLR and BMI values and a lower NLR value in the obese group compared with the normal group. The obese group showed a higher lymphocyte count, thereby confirming the positive correlation of lymphocyte count with BMI.A comprehensive understanding of the mechanisms underlying the relationship between obesity and inflammation may allow developing treatment strategies to reduce the negative effects of obesity.
